# Case-Finding tool for COPD in LMIC (COLA) - translation and cross-cultural adaptation into Brazilian Portuguese language

**DOI:** 10.11606/s1518-8787.2023057004904

**Published:** 2023-09-14

**Authors:** Erika Zavaglia Kabbach, Naiara Tais Leonardi, Trishul Siddharthan, Audrey Borghi-Silva, Jaber Saud Alqahtani, John R Hurst, Renata Gonçalves Mendes

**Affiliations:** I Universidade Federal de São Carlos Department of Physical Therapy São Carlos SP Brazil Universidade Federal de São Carlos, Department of Physical Therapy, São Carlos, SP, Brazil; II University of Miami Medicine Miller School of Medicine Division of Pulmonary, Critical Care and Sleep Miami FL USA University of Miami, Medicine Miller School of Medicine, Division of Pulmonary, Critical Care and Sleep, Miami, FL, USA; III Prince Sultan Military College of Health Sciences Department of Respiratory Care Dammam Saudi Arabia Prince Sultan Military College of Health Sciences, Dammam, Department of Respiratory Care, Saudi Arabia; IV University College London UCL Respiratory London UK University College London, UCL Respiratory, London, UK

**Keywords:** Lung Diseases, Pulmonary Disease, Chronic Obstructive, Mass Screening, Surveys and Questionnaires, Language

## Abstract

**OBJECTIVE:**

To translate and cross-culturally adapt the COPD in Low- and middle-income countries (LMICs) Assessment (COLA) questionnaire into Brazilian Portuguese, a case-finding instrument for chronic obstructive pulmonary disease (COPD).

**METHODS:**

Translation and cross-cultural adaptation were completed in six steps: the original version was translated into Brazilian Portuguese by two native speakers of the target language; the translated versions were synthesized; back-translation was performed by two native speakers of the original language; the back-translation and the Brazilian Portuguese version of the COLA were reviewed and harmonized by an expert committee of specialists; and, then, the pre-final version was tested by 30 health professionals who were asked if the items were clear to understand. The acceptability, clarity, and understandability of the translated version were evaluated. A final review of the questionnaire was produced by the authors and approved by the author of the original questionnaire.

**RESULTS:**

Some idiomatic, semantic, and experiential inconsistencies were identified and properly adjusted. Item 3 was considered the most unclear item (23,3%). Items 7, 8, and 9 presented clarity above 80% (93%, 90%, and 90%, respectively). Suggestions were discussed and incorporated into the tool and COLA was found to be clear and easy to understand.

**CONCLUSIONS:**

The Brazilian version of the COLA was easily understood by healthcare professionals and adapted to Brazilian culture. Translation and cultural adaptation of the COLA instrument into Brazilian Portuguese can be an important case-finding instrument for chronic obstructive pulmonary disease in Brazil.

## INTRODUCTION

Chronic Obstructive Pulmonary Disease (COPD) is a non-communicable chronic respiratory disease representing the third leading cause of death worldwide^1,2^. According to the Global Burden of Disease study, over 90% of morbidity and mortality related to COPD occurs in low- and middle-income countries (LMICs)^3^. Recently, the prevalence of COPD in Brazil was found to be 17%, and it is among the most prevalent diseases globally^4^.

The GOLD Strategy Document has recently outlined, particularly in LMIC, that case-finding and early diagnosis can be goals to reduce early mortality and the impact of COPD worldwide^5^. Currently, COPD remains largely undiagnosed or diagnosed late during the disease^6^. Spirometry is the gold standard for confirming the diagnosis of COPD^7^; however, studies have demonstrated some organizational and technical barriers that lack in LMICs, such as access to diagnostic equipment, trained technicians, and specialized clinicians to interpret the studies^8,9^.

A strategy for the early diagnosis is to achieve more reliable screening for COPD making questionnaires available, which are simple and potentially cost-effective, identifying those at risk of COPD^5^. Some case-finding questionnaires have been developed^10^, comprising self-reported instruments which rely on symptoms and risk factors related to COPD, and have been validated among clinic-based populations with moderately high predictive value. However, these instruments were not designed for different languages and wider use in LMICs, where history of lung disease can differ in several aspects including different kinds of exposure to risk factors and access to healthcare.

In this context, a seven-item questionnaire called COLA (COPD in LMICs Assessment) was developed in the English language by Siddharthan et al.^13^ in a study conducted in Uganda (Nakaseke and Kampala areas), from November 2015 to June 2016. The case-finding tool consisted of respiratory symptoms/functional status, risk factors, and exposure, in addition to age and peak expiratory flow measurements, demonstrating a high discrimination for COPD in a population-based setting^13^. COLA is not a self-administered questionnaire; it is intended to be used by any healthcare professional who is involved in the screening of people at higher risk of COPD according to medical history (target of the tool) as a final user. The potential of different case finding tools in resource-limited settings has been recently published^14^.

Therefore, implementation of COPD case finding strategies are recommended to support the screening of underdiagnosed cases and identification of individuals more susceptible to COPD^8^ and deserve to be adequately tested in real-world settings in LMICs. This study aimed to produce a culturally equivalent version of the COLA questionnaire^13^ in Brazilian Portuguese using the translation and cross-cultural adaptation of the instrument.

## METHODS

### Study Design

This study was conducted at the Cardiopulmonary Laboratory at the Federal University of São Carlos, SP - Brazil. This study was approved by the Research Ethics Committee of UFSCar (CAAE 43115521.0.0000.5504) and all the participants signed an informed consent form before being included in the study. Translation and cross-cultural adaptation of COLA was performed with prior permission from the original author (TS) via e-mail and consisted of six steps^15^: (1) Translation of the original into Brazilian Portuguese; (2) Synthesis of the translated versions; (3) Back-translation; (4) Review and harmonization of the back-translation; (5) Pretesting of final version (6) Final review.

### Participants

A total of 30 healthcare professionals (physiotherapists, physicians, and nurses), members of the Federal University or University Hospital of Sao Carlos, Brazil, were enrolled in the fifth step (test of the pre-final version) of this methodological study. The inclusion criteria were native Brazilians healthy professionals, aged from 25 to 65 years (graduated with some professional background), able to consent. The exclusion criteria were professionals who did not speak Brazilian Portuguese as their native language. The included participants were intentionally invited in-person and an online link to access the COLA questionnaire via Google Forms® platform was sent from July to December 2021.

### Description of the COLA Questionnaire Score

The COLA (COPD in LMICs Assessment) is a case finding multi-domain questionnaire composed of three parts: 1) seven questions about respiratory symptoms, functional status, personal exposures; 2) age; and 3) peak expiratory flow values^13^. Individuals receive 1 point for each of the seven questions (if present), 1 point if the participant is ≥ 55 years of age, 1 point if peak expiratory flow is ranges from 250 to 399 L/min, and 2 points if peak expiratory flow (PEF) is less than 250 L/min. A COLA score ≥ 5 presented the best combination of sensitivity, specificity, and positive predictive value to predict risk for COPD^13^.

### Translation and Cross-cultural Adaptation

The translation and cross-cultural adaptation process were conducted in six steps, following internationally accepted guidelines^15^: The steps are standardized throughout the text and numbered accordingly:

1. Translation of COLA questionnaire from English to Brazilian Portuguese was performed by two professional translators (Translator 1 - T1 and Translator 2 - T2) who were hired for this process. Both were fluent in English and native speakers of Brazilian Portuguese, one of whom had no specific knowledge of health. The translations were completed independently from each other (T1 and T2).
2. Reconciliation and synthesis of translations took place in the second stage, in which the final draft of the translation was revised for any conceptual errors or inconsistencies by both the translators mentioned in Step 1 and by an invited person (medical colleague) who had a good knowledge of both English and Brazilian Portuguese (an observer). The next stage was completed with this version of the questionnaire (V1).
3. In the third step, the questionnaire was back-translated into English by two specialist independent translators, who were paid for the back-translation, both with English as their mother tongue and fluent in Brazilian Portuguese. Both translators were blinded to the study and had no access to the original questionnaire.
4. In the fourth step, an expert committee (Translators of Step 1 and 3; and pulmonary specialists) compared the questionnaire back-translated into English to the original version of the questionnaire and reviewed T1, T2, and V1 on the Brazilian Portuguese version of the questionnaire. Each item, in terms of conceptual (referring to the conceptual formulation of the evaluation), idiomatic (different linguistic expressions), semantic (differences related to the meaning of test), and experiential (related to cultural differences) equivalences were evaluated, giving rise to the pre-final version of the questionnaire. For the semantic and idiomatic analyses, the experts marked the item as unchanged if it was fully similar to the original scale item, as slightly changed if some words of the item were synonymous terms, and as heavily changed if there were words that would change the context of the item and if there were no synonymous terms. The expert committee analyzed whether the content of the items was pertinent to each domain, whether it was appropriate for Brazilian culture, and whether they agreed with the item. Thus, a pre-final version of the questionnaires was reached in an agreed way between all members of the committee (V2).
5. The fifth step consisted of testing the pre-final version. The cognitive debriefing aimed to identify inconsistencies, offering solutions to make questions easier to understand. In this step, acceptability, clarity, and understandability of the pre-final version were assessed in a predetermined convenience sample of 30 healthcare professionals^15^. The sample size was established following the guidelines for the process of cross-cultural adaptation of self-report measures^15^. Participants were instructed to respond to an online version of COLA focusing on the clarity of each item as “Yes, this is clear” or “No, this is not clear.” If an item was unclear, the item should be revised according to comments and suggestions. Unclear item was defined as a percentage below 80% of agreement regarding clarity (positive answer - Yes) for each item of the questionnaire, considering the response of all 30 healthcare professionals. At the end, individuals were asked to make a general comment about overall acceptability, understandability, and clarity to establish the final version of the questionnaire in Brazilian Portuguese. All comments were recorded on a specific form. In addition, six random COPD patients (tool target) attending the Laboratory of Cardiopulmonary Physiotherapy were informally consulted for an opinion about the tool with no negative comments to worry about.
6. A final review was performed according to inconsistencies pointed out by the sample of healthcare professionals in the pre-final version. The instrument was analyzed item by item. The cognitive debriefing findings were discussed by the authors of this study and expert committee and the relevant changes were made. After corrections, the original author verified the agreement with the version. This step resulted in a final version (FV), the Brazilian version of the COLA.

### Statistical Analysis

In this translation and cross-cultural adaptation study, the descriptive statistical analysis of sociodemographic and clinical characteristics of participants included was performed by calculating absolute and relative frequency for qualitative variables and central tendency (mean, standard deviation, median, quartiles, minimum, and maximum) for quantitative variables. The cultural adequacy of the questionnaire was assessed by the clarity of each item, considering the answers from 30 healthcare professionals as follows: Percentage of clarity = number of “Yes, this is clear’’ (1) x 100/ 30 to each item. For clarity below 80%, the specific item was revised and changes were provided according to the participants’ comments and suggestions.

## RESULTS

Translation and cultural adaptation processes were completed following the method outlined above. Box 1 summarizes the results of the synthesis of the translated, back-translated, pre-final, and final version.

**Box 1:** Original, translated, back-translated, pre-final, and final version of the COLA.

Original version	Synthesis of the translations (version 1 – V1)	Back-translated version	Pre-Final Version (version 2 – V2)	Final Version (FV)
Item 1. Have you had whistling/wheezing in chest in last 12 months?	*Durante os últimos 12 meses, você sentiu assobio/chiado no peito?*	In the past 12 months, have you felt whistling/wheezing in the chest?	*Nos últimos 12 meses, você sentiu chiado no peito?*	*Nos últimos 12 meses, você sentiu chiado no peito?*
Item 2. Have you ever been woken up from sleep by wheezing?	*Alguma vez você acordou devido ao chiado?*	Have you ever woken up due to wheezing?	*Você tem acordado devido ao chiado no peito?*	*Você tem acordado devido ao chiado no peito?*
Item 3. Have you brought up phlegm from your chest on most days or nights of the week during at least 3 months in at least 2 years?	*Você eliminou catarro do peito na maioria dos dias ou noites da semana, durante pelo menos 3 meses, em pelo menos 2 anos consecutivos?*	Did you cough up phlegm the majority of nights of the week for at least three months in at least two consecutive years?	*Você tem expectorado catarro do peito na maioria dos dias da semana por pelo menos 3 meses nestes últimos dois anos?*	*Você tem expectorado/cuspido catarro do peito frequentemente (dias e/ou noites), por pelo menos 3 meses no ano, por pelo menos 2 anos seguidos?*
Item 4. In the past 12 months, have you had to miss work or have your daily activities been impeded because of your respiratory problems?	*Nos últimos 12 meses, você teve que faltar ao trabalho, ou teve suas atividades diárias afetadas devido aos seus problemas respiratórios?*	In the past 12 months, have you had to miss work or had your daily activities affected due to your respiratory problems?	*Nos últimos 12 meses, você teve que faltar do trabalho, ou teve suas atividades diárias afetadas devido aos seus problemas respiratórios?*	*Nos últimos 12 meses, você teve que faltar do trabalho, ou teve suas atividades diárias afetadas devido aos seus problemas respiratórios?*
Item 5. In the past 12 months, have you been hospitalized because of respiratory problems?	*Nos últimos 12 meses, você foi hospitalizado/a devido a problemas respiratórios?*	In the past 12 months, were you hospitalized due to respiratory problems?	*Nos últimos 12 meses, você foi hospitalizado/a devido aos problemas respiratórios?*	*Nos últimos 12 meses, você foi hospitalizado/a devido aos problemas respiratórios?*
Item 6. Do you currently smoke?	*Você fuma atualmente?*	Do you currently smoke?	*Você fuma atualmente?*	*Você fuma atualmente?*
Item 7. Do you use biomass fuel daily?	*Você usa combustível de biomassa (lenha, carvão, madeira ou outro) diariamente?*	Do you use biomass fuel (firewood, coal, wood or other) daily?	*Você tem contato com a fumaça da queima da lenha ou carvão diariamente?*	*Você tem contato com a fumaça da queima da lenha, carvão, cana de açúcar ou outro material (biomassa) diariamente?*
Item 8. Age score:	*Faixa etária*	Age group	*Faixa etária*	*Faixa etária:*
< 55 years;	*< 55 anos*	< 55 years	*< 55 anos*	*menor que 55 anos; maior ou igual a 55 anos*
≥ 55 years	*≥ 55 anos*	≥ 55 years	*≥ 55 anos*	
Item 9. Peak expiratory flow score:	*Pontuação do pico do fluxo expiratório:*	Peak expiratory flow score	*Pontuação do pico do fluxo expiratório:*	*Pontuação do pico de fluxo expiratório:*
≥ 400L/min;	*≥ 400 L/min*	≥ 400 L/min	*≥ 400 L/min*	*maior ou igual a 400L/min,*
250–399 L/min;	*250–399 L/min*	250–399 L/min	*250–399 L/min*	*250 a 399L/min;*
< 250L/min	*< 250 L/min*	< 250 L/min	*< 250 L/min*	*menor que 250L/min*

As expected, some idiomatic, semantic, and experiential inconsistencies were identified and properly adjusted during the process. In part 1 (Symptoms/Functional Score), Item 3 (*Have you brought up phlegm from your chest on most days or nights of the week during at least 3 months in at least 2 years?)* and Item 7 (*Do you use biomass fuel daily?)* were those with the greatest need for adjustments. Therefore, adjustments were made to improve understanding as suggested by healthcare professionals: Item 3, we have changed “*most days or night of the week*” to “*frequently (days and/or nights)*” and added the term “*consecutive*” to the end of the sentence (“*for at least 2 consecutive years*”). Item 7 was adjusted with more common terms of the Brazilian population and examples were added to the term biomass.

Regarding part 2 (age score) and 3 (peak expiratory flow score), there were suggestions to eliminate the original mathematical signs (< and ≥) and describe their meanings to avoid misinterpretation.

As mentioned above, 30 healthcare professionals were included for the pre-final version test step and the characteristics of the participants are shown in Table 1. Most participants were physical therapists (83.3%) from the cardiopulmonary field of expertise (43%) with a mean of 12.23 ± 6.57 years of professional experience.

**Table 1:** Characteristics of participants in the pre-final version test step (n = 30).

Health Professionals, n = 30	
Age (years)	35 ± 66
Occupation	
Physical Therapist	25 (83.3)
Physician	3 (10.0)
Pharmacist	1 (3.3)
Physical Education	1 (3.3)
Field of expertise	
Cardiopulmonary	13 (43)
Intensive Therapy	2 (6.7)
Primary Care	1 (3.3)
Other health occupation	14 (46.7)
Professional experience (years)	12.23 ± 6.57

Data are presented as n (%) or mean ± SD.

The result of this step demonstrated that the most assigned “unclear” item was Item 3 (23,3%), whereas other items of COLA presented a clarity higher than 80% (Table 2). Although items 7, 8, and 9 presented clarity above 80% (93%; 90%, and 90%, respectively), the authors also considered the pertinent comments and suggestions from healthcare professionals for the final version (Box 2).

**Table 2:** Items from the pre-final version considered clear by the participants.

Item	Clear, n (%)	Unclear, n (%)
1	30 (100)	0 (0)
2	26 (86.7)	4 (13.3)
3	23 (76.7)	7 (23.3)
4	30 (100)	0 (0)
5	30 (100)	0 (0)
6	30 (100)	0 (0)
7	28 (93.3)	2 (6.7)
8	27 (90)	3 (10)
9	27 (90)	3 (10)

**Box 2:** Translated and culturally adapted version of COLA questionnaire (COLA Questionário) into Brazilian Portuguese.

*Pontuação funcional e de sintomas*	
*Nos últimos 12 meses, você sentiu chiado no peito?*	1
*Você tem acordado devido ao chiado no peito?*	1
*Você tem expectorado/cuspido catarro do peito frequentemente (dias e/ou noites), por pelo menos 3 meses no ano, por pelo menos 2 anos seguidos?*	1
*Nos últimos 12 meses, você teve que faltar do trabalho, ou teve suas atividades diárias afetadas devido aos seus problemas respiratórios?*	1
*Nos últimos 12 meses, você foi hospitalizado/a devido a problemas respiratórios?*	1
*Você fuma atualmente?*	1
*Você tem contato com a fumaça da queima da lenha, carvão, cana de açúcar ou outro material (biomassa) diariamente?*	1
*Pontuação de faixa etária*	
*Menor que 55 anos*	0
*Maior ou igual a 55 anos*	1
*Pontuação do pico do fluxo expiratório*	
*Maior ou igual a 400 L/min*	0
*250–399 L/min*	1
*Menor que 250 L/min*	2

## DISCUSSION

This study described the translation and cross-cultural adaptation of a case-finding instrument to COPD, the COLA questionnaire, into Brazilian Portuguese. The translated and adapted questionnaire will support the identification of cases more susceptible to COPD. The main result of this study was that the Brazilian version of COLA was successfully adapted to the Brazilian culture and was easily understood by healthcare professionals.

The translation and cross-cultural adaptation aimed to preserve the intent of the original items of the questionnaire while capturing the linguistic nuances within a Brazilian population. Due to the whole process that we described, we considered acceptable the content and format adjustments in the Brazilian Portuguese version. The healthcare professionals also considered most of the items clear for application in the population.

Several case-finding instruments have been validated in high-income settings^10^. These instruments comprise information on symptoms and risk factors related to COPD and have been validated among populations based on clinics with high predictive value. Yawn et al^12^ developed a five-item Lung Function Questionnaire (LFQ) including age, smoking status, and respiratory symptoms. LFQ’s accuracy, sensitivity, and specificity were 0.72, 73.2%, and 58.2%, respectively. The authors found that participants above the age of 50 years had the highest odds for having COPD (OR = 3.32, 95%CI = 1.87–5.90), followed by dyspnea (OR = 1.99, 95%CI = 1.22–3.27) and smoking history (OR = 1.81, 95%CI = 1.33–2.88).

Martinez et al.^11^ developed the CAPTURE tool (COPD Assessment in Primary Care to Identify Undiagnosed Respiratory Disease and Exacerbation Risk), a simple, five-item, patient completed questionnaire, followed by peak expiratory flow (PEF) analysis, to identify undiagnosed cases of COPD in primary care. The questionnaire plus PEF was validated and exhibited an accuracy of 0.91 to identify patients in need of further diagnostic evaluation for COPD, with a sensitivity and specificity of 89.7% and 93.1%, respectively. Both the LFQ and CAPTURE questionnaires were validated in a primary care, clinic-based sample in a high-income country setting.

The COLA instrument exhibited a cross-validated AUC of 0.83 (95%CI 0.78–0.88) with a positive predictive value of 50% and a negative predictive value of 96% with a score greater or equal to five^13^. The choice of the established threshold was associated with high specificity (99%) and lower sensitivity (19%) to reduce health system costs from false-negative, which is important in low-and middle-income settings. Despite achieving a satisfactory diagnostic accuracy, as evidenced by an AUC of 0.74 (95%CI 0.67–0.81)^13^ when incorporating age and seven questions without PEF into the model, further investigation is required to assess its sensitivity and specificity. Thus, COLA in its original format requires PEF testing, and there is no cutoff point to discriminate patients at risk for COPD without PEF. Despite that, although PEF is an effort dependent maneuver and requires some additional training, it is an inexpensive and easy-to-use measure. Thus, COLA may be used by healthcare professionals in clinical and community settings.

To avoid any misinterpretation, it is important to distinguish between screening measures and case-finding in practical applications. Screening involves testing a large number of individuals to identify cases, whereas case-finding, focuses on specific subgroups of individuals at a higher risk, such as symptomatic individuals or those exposed to risk factors^20^. Health community team programs designed to target populations with trained healthcare professionals, mainly in the primary care, may adequately identify patients at-risk for COPD and refer to diagnostic spirometry to provide adequate and early assistance to underdiagnosed patients and improve clinical outcomes and prognosis.

Recently, the discriminative accuracy of CAPTURE, COLA-6, and LFQ have been reported in three diverse LMIC settings^14^. The questionnaires presented generally similar performance and were feasible to deliver by trained research staff. Among the three questionnaires, COLA is the only tool developed for low- and middle-income countries and validated in both urban and rural community settings^13^. COLA also presents an even more positive aspect by including issues related to exposure to biomass fuel. Approximately 15% of the Brazilian population lives in rural areas, and some states in the North and Northeast present 30% to 40% of the population in these areas^21^, favoring the use of biomass burning. In a study conducted in Brazil^22^, COPD was diagnosed in 47 of 160 women exposed to wood-burning stoves equal to or greater than 80 hours per year and for at least 10 years. Thus, the choice to translate and adapt the COLA to Brazilian Portuguese was based on the characteristics of the target population and performance and feasibility of the instrument.

COPD continues to be both underdiagnosed and undertreated^16^. Recently, Lamprecht et al.^17^ analyzed the underdiagnosis of COPD and its determinants in national and international surveys of general populations (44 sites from 27 countries and 30,874 participants). They found that 81.4% of COPD cases were underdiagnosed; there was considerable variation across sites; and underdiagnosis was associated with men, younger age, never and current smoking, poor education, no previous spirometry, and lower airflow limitation^17^.

According to the American Thoracic Society Workshop Report^8^, there is emerging evidence for the use of questionnaires and PEF testing to screen individuals with COPD, as simpler and less costly alternatives to identify patients who will need spirometry confirmation. However, there is still a need to test them in real-world settings in low-and middle-income countries (LMICs)^8^. Thus, this study offers an opportunity to collaborate with this challenge and strategy, considering that, in Brazil, the prevalence of COPD was 17% in the study by Cruz and Pereira^4^, which is higher than the estimated 11.4% for the world population. In addition, PLATINO studies^18,19^presented important evidence of underdiagnoses, with a rate in new COPD cases identified after nine years of follow-up in São Paulo, Brazil, of 70.0%^18^.

Although COPD mortality and morbidity rates were reduced in Brazil from 2000 to 2016 in regions with higher socioeconomic indices, the North and Northeast regions showed an increase in these rates^23^. Furthermore, COPD was responsible for the increase of disability-adjusted life-years (DALYs) with increasing age^24^, which highlights the ageing process, and the epidemiological transition of non-communicable diseases present in developing countries, such as Brazil.

Thus, a simpler and less costly early diagnostic approach is required in Brazil, allowing improvement in access and quality of care across the continuum of the disease, especially in unfavorable socioeconomic regions. This may have a potential impact on individual quality of life and in the setting of public health^25^. Thus, COLA is a promising and useful tool for clinicians and researchers to identify patients at-risk for COPD and refer to diagnostic spirometry to provide adequate and early assistance to underdiagnosed patients and improve clinical outcomes and prognosis.

Therefore, in this study, COLA was translated and culturally adapted for use in Brazil and is adequate for the next steps of validation. For consistent applications of COLA in the Brazilian population, further studies should be conducted, evaluating the discriminative accuracy of this instrument in different regions of Brazil, including urban and rural populations, to determine its feasibility and clinical applicability, in addition to its possible economic importance. Further longitudinal studies will also be needed to assess the contribution of case-finding questionnaires, such as COLA, in reducing underdiagnosed COPD and its complications. Unanswered questions remain regarding the feasibility of questionnaire-based approaches to COPD screening in routine healthcare settings, and the benefits to the individual and society of COPD case-findings.

## CONCLUSION

Translation and cross-cultural adaptation of the COLA instrument (COLA Questionário) into Brazilian Portuguese was performed, providing an important case-finding instrument to facilitate early diagnosis of COPD in Brazil. Further studies should focus on the discriminative accuracy of COLA in identifying patients at high risk of COPD in the Brazilian population.
